# Diagnosis of Lung Cancer by Fractal Analysis of Damaged DNA

**DOI:** 10.1155/2015/242695

**Published:** 2015-10-11

**Authors:** Hamidreza Namazi, Mona Kiminezhadmalaie

**Affiliations:** ^1^Department of Mechanical Engineering, Faculty of Engineering, Universiti Malaysia Sarawak, 94300 Kuching, Sarawak, Malaysia; ^2^Faculty of Biosciences & Medical Engineering, Universiti Teknologi Malaysia, 81310 Johor Bahru, Malaysia

## Abstract

Cancer starts when cells in a part of the body start to grow out of control. In fact cells become cancer cells because of DNA damage. A DNA walk of a genome represents how the frequency of each nucleotide of a pairing nucleotide couple changes locally. In this research in order to study the cancer genes, DNA walk plots of genomes of patients with lung cancer were generated using a program written in MATLAB language. The data so obtained was checked for fractal property by computing the fractal dimension using a program written in MATLAB. Also, the correlation of damaged DNA was studied using the Hurst exponent measure. We have found that the damaged DNA sequences are exhibiting higher degree of fractality and less correlation compared with normal DNA sequences. So we confirmed this method can be used for early detection of lung cancer. The method introduced in this research not only is useful for diagnosis of lung cancer but also can be applied for detection and growth analysis of different types of cancers.

## 1. Introduction

Cancers are caused by uncontrollable growth of cells which do not die. Normal cells in the body grow, divide, and finally die (apoptosis) in an orderly path. When the death process of cells breaks down, cancer starts. In case of cancer, cells continue to grow and divide instead of having a programmatic death which results in a bunch of abnormal cells growing out of control.

Lungs are spongy organs in the chest which take in oxygen and release carbon when human inhales and exhales, respectively. Lung cancer begins in the lungs. Lung cancer is a dominant type of cancer which kills many people every year compared to other types of cancer.

When the cell's gene cannot correct the DNA damage, the lung cancer appears. Inhaling carcinogenic substances is the main reason of lung cancer.

For years some methods have been investigated in order to diagnose the lung cancer. Most of these methods are based on medical theories. Among these methods, employing computed tomography (CT) image analysis is more dominant. By computed tomography of chest Mets et al. derived and validated a model of lung cancer which studies the coronary and aortic calcium volume in lung [1]. Veronesi et al. analyzed computed tomography images of lung in case of smokers and former smokers in order to detect the lung cancer [2]. In another work Jiménez-Bonilla et al. worked on diagnosis of recurrence and assessment of postrecurrence survival in patients with extra cranial non-small cell lung cancer using 18F-FDG PET/CT [3]. See also [4–6]. On the other hand, some researchers have worked on analysis of patients' DNA for diagnosis lung cancer. An et al. detected tumor-associated aberrant hyper methylation of the* p16* gene in DNA extracted from plasma. Using a modified seminested methylation-specific PCR, they did their experiments on 105 non-small cell lung cancer patients and 92 matched tumor DNA samples [7]. In a recent research, Jelovac et al. detected PIK3CA DNA mutation in plasma of a patient with breast and lung cancers [8]. In another extensive work, Diehn et al. employed deep sequencing for detection of circulating tumor DNA in non-small cell lung cancer [9].

Beside some works done on the prediction and analysis of lung cancer from biological point of view, there are few researches being reported which use mathematical models for diagnosis of lung cancer. McCulloch et al. used mathematical model for developing a model-based CAD algorithm which capture scanner physics and anatomic information. Their model uses multiple segmentation algorithms in order to extract structures in the lungs. Also, they proposed a selection framework which was based on Bayesian statistical model in order to determine the probability of different anatomical events throughout the lung [10]. Kang et al. constructed a diagnostic and prognostic mathematical model of lung cancer. In fact this model integrates let-7 and miR-9 expression into a signalling pathway in order to generate an in silico model for the process of epithelial mesenchymal transition (EMT). They used this model for diagnostic and prognostic biomarkers in lung cancer [11]. In a recent work, Hndoosh suggested a fuzzy mathematical model for detection of lung cancer using a multi-NFclass with confusion fuzzy matrix for accuracy [12].

Fractals are scale-invariant geometric objects. A scale-invariant object can be self-similar or self-affine. A self-similar object is a union of rescaled copies of itself which is isotropic or uniform in all directions. But in case of self-affine objects, the mechanism is anisotropic or dependent on the direction. Regular fractals have higher self-similarity, but random fractals have a weaker self-similarity.

The class of regular fractals includes many familiar simple objects, such as line intervals, solid squares, and solid cubes, and also many irregular objects. The scaling rules are characterized by “scaling exponents” (dimension). “Simple” regular fractals have integer scaling dimensions. Complex self-similar objects have noninteger dimension. Therefore, it is completely incorrect to define fractals as geometric objects having “fractional” (noninteger) dimension. Fractals may be defined as geometric objects whose scaling exponent (dimension) satisfies the Szpilrajn inequality:

(1) $$\begin{array}{c} ℵ\geq D_{T}, \end{array}$$

where *ℵ* is the scaling exponent (dimension) of the object and *D*_*T*_ is its topological dimension, that is, Euclidean dimension of units from which the fractal object is built. For example, in case of Brownian motion: the path of a particle, a line of dimension one, traveling for a long time over a plane region, eventually covers the entire plane, an entity of dimension two [13].

In case of multifractal system a single fractal dimension cannot describe its dynamics. In this case, a continuous spectrum of exponents is needed [14].

We deal with many multifractal systems in nature such as fully developed turbulence and heartbeat dynamics. In case of using fractal for detection of lung cancer, limited works have been reported which are based on analysis of tumor's shape using fractal dimension. Miwa et al. found out that the d-FD of intratumoral heterogeneity of FDG uptake can help to differentially diagnose malignant and benign pulmonary nodules. The SUVmax and d-FD obtained from FDG-PET images provide different types of information that are equally useful for differential diagnoses [15]. Lee et al. reported that fractal dimension of carcinoma epithelial architecture can assist in differentiating adenocarcinoma (ADC) from squamous cell carcinoma (SCC) of the lung [16].

In spite of all of these works, no work has been reported which analyses the complexity and correlation of damaged DNA. In this paper we use the concept of fractal dimension and the Hurst exponent in order to analyse the DNA sequences. In order to do this task first we illustrate DNA walk as a random walk and then by introducing the spectra of fractal dimension and the Hurst exponent we compute these parameters for DNA walks extracted from DNA sequences of patients with lung cancer. The multifractality and correlation of patients' DNA walk are discussed in detail.

## 2. DNA and Random Motion

A DNA sequence of chromosome in the cell's nucleus is a combination of four letters. These letters, A, C, G, and T, are the bases adenine, cytosine, guanine, and thymine, respectively. For instance, a DNA sequence is

 …GTCAGAGCCTATCGTTACG…

This string can be written in the form of numbers by assigning 1 for T, 2 for A, 3 for C, and 4 for G, so

 …4132424331213411234…

In fact, the DNA sequence is the root for development of a complete organism.

For years many mathematical methods have been developed in order to study the nature of DNA sequences.

DNA walk plotting is a popular method which generates a planar trajectory of DNA sequences. In this method first the DNA text is converted to a binary sequence and then the DNA walk plot is defined by the cumulative variables [17]. By this consideration, DNA walk can be considered as a random walk (Brownian motion) where each point in the plot can deflect up or down in nucleotide distance.

DNA sequence can be defined by two of six possible combinations which are purines (A+G), pyrimidines (C+T), Imino (A+C), Keto (G+T), Weak (A+T), and Strong (G+C). Combination of pyrimidine tract with purines tract is long known for analysis of DNA [18]. In this research we chose this combination as it helps in better detection of the long dependence property in DNA sequences (see [17, 19]).

**Figure 1:**
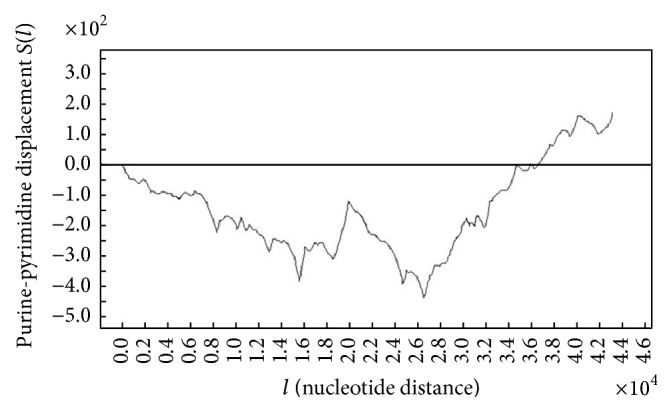
The plots of DNA walk of original sequence.

Figure 1 shows the one-dimensional DNA walk plot using purine-pyrimidine binary rule. This rule changes purines (A/G) to −1 and pyrimidines (C/T) to +1.

In the next section by introducing the Hurst exponent we discuss the correlation of the random walks and in special case the DNA walks.

## 3. Hurst Exponent and Type of Motion

**Figure 2:**
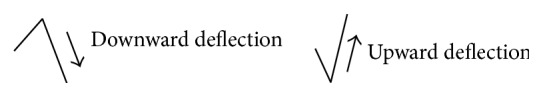
Upward and downward deflection in the DNA walk from one point to the next point.

In order to analyse the behaviour of a DNA walk, the direction of fluctuation (deflection) from one point to the next point [20] and in a bigger view the correlation of walk should be considered (Figure 2). This behaviour can be studied by computing the Hurst exponent. The Hurst exponent is an indicator of the long term memory of the process. In fact, it is the measure of the predictability of the DNA walk.

The value of the Hurst exponent can be between 0 and 1, where the value that it gains in each moment determines the behaviour of the next deflection in the random walk.

Each point in the DNA walk can deflect up or down based on the binary map of the DNA sequence. In case of no correlation between the points in walk, the Brownian motion is dominant. Otherwise, long term memory and accordingly fractional Brownian motion define the walk. These two conditions can be characterized by different values of the Hurst exponent. When *H* = 0.5, the process is Brownian motion and when 0 < *H* < 1  (*H* ≠ 0.5) the process is fractional Brownian motion. In case of Brownian motion, *H* = 0.5, the process is considered to be truly random (e.g., Brownian motion). It means that there is absolutely no correlation between any values of the process and it is hard to predict the future of process. The analysis of the Hurst exponent for fractional Brownian motion can be categorized in two ranges. Firstly, if the Hurst exponent has a value between 0 and 0.5, it means that the process is antipersistent; that is, the trend of the process at the next instant will be opposite to the trend in the previous instant. Secondly, a value of *H* between 0.5 and 1 means that the process is persistent; that is, the trend of the process at the next instant will be the same as the trend in the previous instant.

In this research, we compute the value of the Hurst exponent for damaged DNA walk and compare its values with the normal DNA walk. This comparison helps us to find out about the correlation and predictability of damaged versus normal DNA walk.

There are different methods which have been developed to estimate the value of *H*. Rescaled range analysis (*R*/*S*) and DFA are two main methods for estimation of the Hurst exponent. By the initial analysis of computed Hurst exponent of DNA walks we found out that even if *R*/*S* method shows higher values of Hurst exponent than DFA, the standard deviation has lower values for *R*/*S*, and then the confidence intervals are narrower. Thus in our case *R*/*S* method is more precise. It is noteworthy that both methods show closer results as the DNA sequence becomes longer.

So in this research we employ *R*/*S* analysis method for computing the Hurst exponent which is described in the next section using a sample.

## 4. Rescaled Range Analysis of the DNA Sequence

*R*/*S* analysis is described in many literatures as a famous method for calculating the Hurst exponent of time series. So, by employing this method, the values of the Hurst exponent can be computed for the DNA walk. The same method used in case of time series can be applied to DNA sequence. The calculations are explained here through a sample. Consider

(2) $$\begin{array}{c} X\left(s,l\right)=\overset{s}{\underset{u=1}{\sum }}\left\{\xi \left(u\right)-{\langle \xi \rangle }_{l}\right\}. \end{array}$$

In (1), *s* is a letter on the sequence which has *l* letters long, and

(3) $$\begin{array}{c} {\langle \xi \rangle }_{l}=\frac{1}{l}\overset{l}{\underset{s=1}{\sum }}\xi \left(s\right). \end{array}$$

In an application sample brought in Table 1, the sum of movements is computed as

(4) $$\begin{array}{c} \overset{l}{\underset{s=1}{\sum }}\xi \left(s\right)=9521+6398-9487-6170=262. \end{array}$$

So,

(5) $$\begin{array}{c} {\langle \xi \rangle }_{l}=\frac{1}{l}\overset{l}{\underset{s=1}{\sum }}\xi \left(s\right)=\frac{262}{31576}=0.008297\approx 0. \end{array}$$

Thus,

(6) $$\begin{array}{c} X\left(s,l\right)=\overset{s}{\underset{u=1}{\sum }}\left\{\xi \left(u\right)-{\langle \xi \rangle }_{l}\right\}\approx \overset{s}{\underset{u=1}{\sum }}\left\{\xi \left(u\right)-0\right\}=\overset{s}{\underset{u=1}{\sum }}\left\{\xi \left(u\right)\right\}. \end{array}$$

By conversions of adequate letter we define

(7) $$\begin{array}{c} R\left(l\right)=\max X\left(s,l\right)-\min X\left(s,l\right), \end{array}$$

(8) $$\begin{array}{c} S={\left[\frac{1}{l}\overset{l}{\underset{s=1}{\sum }}{\left\{\xi \left(s\right)-{\langle \xi \rangle }_{l}\right\}}^{2}\right]}^{1/2}. \end{array}$$

From (6),

(9) $$\begin{array}{c} R\left(l\right)=\max\overset{s}{\underset{u=1}{\sum }}\xi \left(u\right)-\min\overset{s}{\underset{u=1}{\sum }}\xi \left(u\right). \end{array}$$

From Figure 1,

(10) $$\begin{array}{c} R\left(l\right)=205-\left(-430\right)=635. \end{array}$$

From (5) and (8)

(11) $$\begin{array}{c} S={\left[\frac{1}{l}\overset{l}{\underset{s=1}{\sum }}{\left\{\xi \left(s\right)-0\right\}}^{2}\right]}^{1/2}={\left[\frac{1}{l}\overset{l}{\underset{s=1}{\sum }}{\left(\xi \left(s\right)\right)}^{2}\right]}^{1/2}\approx 1. \end{array}$$

So

(12) $$\begin{array}{c} \frac{R}{S}\approx R\left(l\right)={\left(\frac{l}{2}\right)}^{H}. \end{array}$$

Consequently,

(13) $$\begin{array}{c} H=\frac{\log R\left(l\right)}{\log\left(l/2\right)}=\frac{\log635}{\log\left(31576/2\right)}\approx 0.668. \end{array}$$

Based on the last discussion, the value of *H* suggests that there exists good persistence in the DNA walk as it is between 0.5 and 1.

Some of published values of *H* for DNA sequences are brought in Table 2.

As it can be seen in Table 2, there are good correlations in all cases for normal DNA.

In this research *R*/*S* values are computed for *l*, *l*/2, *l*/4,…, and *l*/2^*n*^. Then, for each division of *l*, the average value of *R*/*S* is computed again. The value of the Hurst exponent is obtained by computing the slop of linear regression line in log⁡(*R*/*S*) versus log⁡*l* plot. In this research we calculate the Hurst exponent in different segment of DNA walk and report a signal-shaped plot for it not only an average value. Using this method we are able to talk about the memory and predictability in the DNA walk.

## 5. Spectra of Fractal Dimension

In this section we use the concept of fractal dimension for computing the complexity of DNA walk. In order to use this measure we consider the equations in the work done by Kulish et al. [21] by converting in case of DNA walk instead of time series.

In case of DNA walk with *ξ*_max⁡_ and *ξ*_min⁡_, by dividing the total range in *N* bin,

(14) $$\begin{array}{c} N=\frac{\xi_{\max}-\xi_{\min}}{\delta \xi }. \end{array}$$

The probability that the value falls into the *i*th bin of size *δξ* is computed as

(15) $$\begin{array}{c} w_{i}=\underset{N\to \infty }{\lim}\frac{N_{i}}{N}, \end{array}$$

where *N*_*i*_ equals the number of items the value falls into the *i*th bin. In case of a DNA walk,

(16) $$\begin{array}{c} w_{i}=\underset{l\to \infty }{\lim}\frac{s_{i}}{l}, \end{array}$$

where *s*_*i*_ is letter in the *i*th bin in the entire sequence of length *l*.

The Renyi entropy for the probabilities of letters of order *q* is

(17) $$\begin{array}{c} E_{q}=\frac{1}{1-q}\log_{2}\overset{N}{\underset{i=1}{\sum }}w_{i}^{q}. \end{array}$$

Note that for *q* → 1

(18) $$\begin{array}{c} E_{1}=-\overset{N}{\underset{i=1}{\sum }}w_{i}\log w_{i}. \end{array}$$

The fractal dimensions for a DNA walk are defined as

(19) $$\begin{array}{c} {ℵ}_{q}=\underset{\delta \xi \to 0}{\lim}\frac{1}{q-1}\frac{\log\sum_{i=1}^{N}w_{i}^{q}}{\log\delta \xi }, \end{array}$$

where −*∞* < *q* < +*∞*. In case of a self-similar series with equal probabilities, *w*_*i*_ = 1/*N* is the same for the entire of series. In this case, (19) yields *ℵ*_*q*_ = *ℵ*_0_ for all values of *q*.

For a given DNA walk, fractal dimension (*ℵ*_*q*_) which is computed from (19) stands for the probability distribution of walk. A bigger value of *ℵ*_*q*_ corresponds to the more complex (less predictable) DNA walks which have shaper fluctuations. Also, when the range of fractal dimension variation is wider, the DNA walk is more fractal. It is clear that the zero range stands for self-similar fractals.

Considering the unexpectedness, DNA walk with steeper spectra has more unexpected values. On the other hand, flatter spectra stand for less unexpectedness [13].

## 6. Result and Discussion

In this section we compute the Hurst exponent and fractal dimension for DNA walks in case of different subjects and compare the results for diagnosis of skin cancer.

### 6.1. Data Collection

Scientists have found that tumors shed nucleic acids (DNA or RNA) into the blood stream. So, the plasma can be used as the source of tumor DNA [22]. Scientists believe that plasma DNA is of tumor origin as its genetic alterations are similar to the corresponding primary tumors [23].

In this research, blood plasma samples were collected for genetic testing from 50 patients with histologically confirmed lung cancer (group 1) (non-small cell carcinoma) and 50 healthy control individuals (group 2). In each group, 25 subjects are men and 25 subjects are women and all of them are 32 years old. It is noteworthy that all patients were smoker. Patients did not receive chemotherapy or radiotherapy before their recruitment. Healthy individuals with no prior history of cancer were recruited from the staffs in our institution. Information on smoking habits was collected by means of self-reporting.

In this research we employed the similar methodology employed by Weber et al. [24] for collection of DNA samples. In this research we used 2 mL of the plasma. We prepared Proteinase K with two wash buffers (WBI) in DNA sample preparation kit. Then, we mixed the plasma with 260 *μ*L Proteinase K and 2.1 mL DNA PBB (binding buffer) and incubated it at room temperature for 25 minutes. After that we mixed 500 *μ*L isopropanol with the lysate and then transferred it into the High Pure Extender Assembly. Then, these assemblies were centrifuged at 4000 ×g for 1 min. The DNA was eluted in 100 *μ*L DNA EB (elution buffer). The extraction yields high quality DNA suitable for further analyses.

All procedures were approved by the Internal Review Board of the University and the approval for experimentation involving human subjects was issued by Sarawak General Hospital and the university. It is noteworthy that the identity of all subjects remains confidential.

### 6.2. Data Analysis

In order to do the analyses at first the data were preprocessed to homogenize the data set. A program was written in MATLAB to generate the DNA walk for the sequences. This program maps the DNA sequences to DNA walk using the method discussed in Section 2. After this being established, the DNA walk is analysed by computing the Hurst exponent and fractal dimension based on the methods brought in Sections 4 and 5. These analyses are done using a program written in MATLAB.

**Figure 3:**
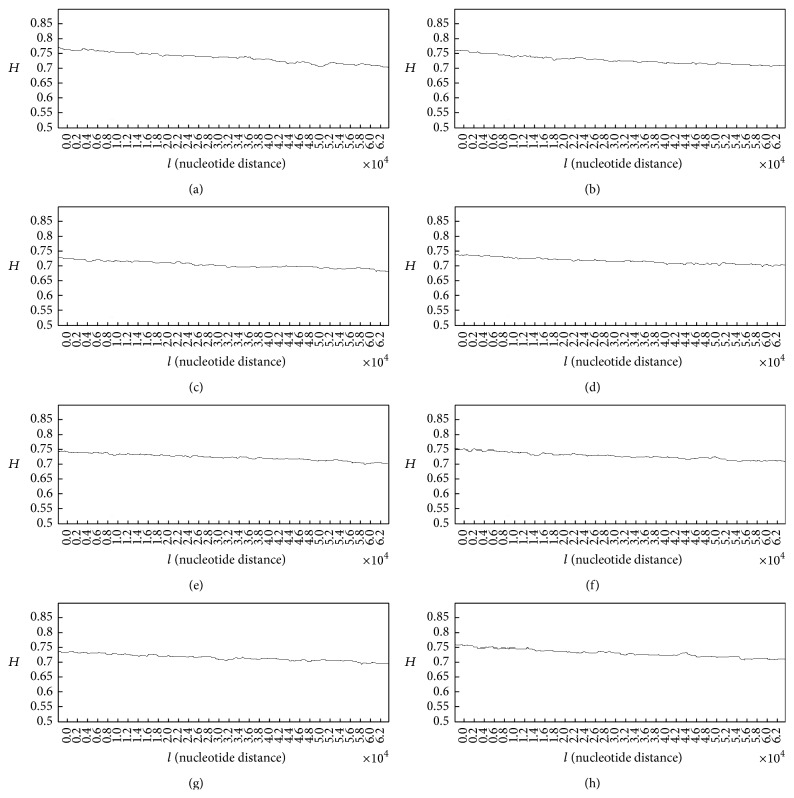
Generated Hurst exponent plots for DNA walks of 8 healthy subjects.

Here, we bring some of the generated plots for the Hurst exponent and fractal dimension spectra in case of healthy subjects and patients with lung cancer.  Figure 3 shows the Hurst exponent plots for the DNA walks in case of healthy subjects.

As it can be seen in plots (a) to (h) (Figure 3) the overall behaviour of the Hurst exponent variations is decreasing behaviour as its value tends to *H* = 0.5. This behaviour stands for the fact that memory of DNA walk is decreasing in the genome. The small upward deflections seen in the plots stand for the small increases in the genome's memory. It is clear that by decreasing the value of *H* (getting closer to 0.5) and accordingly the memory of genome, the predictability of DNA walk is decreasing. Having the value of *H* in all plots in the range of 0.5 < *H* < 1 stands for the fact that there are good correlations in the DNA walks. The averaged value of the Hurst exponent for 50 subjects was computed as 0.726.

**Figure 4:**
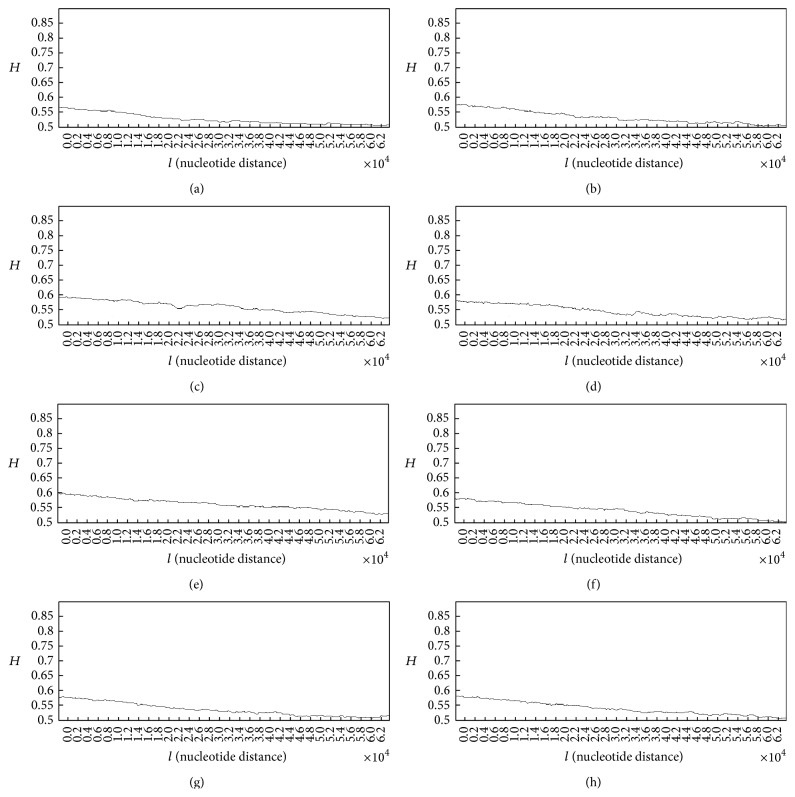
Generated Hurst exponent plots for damaged DNA walks of 8 patients with lung cancer.

In case of patients with lung cancer, some of the generated Hurst exponent plots for damaged DNA walks are shown in Figure 4.

The analysis of plots for damaged DNA walks shows almost similar behaviour to the Hurst exponent plots for the normal DNA walks. As it can be seen in plots (a) to (h) (Figure 4) in overall the Hurst exponent variations tend to *H* = 0.5. But as it is clear, in case of damaged DNA, variations of the Hurst exponent show steeper behaviour than cases that belong to normal DNA walks in Figure 3. Like plots in Figure 3, the small upward deflections seen in the plots stand for the small increases in the genome's memory. It is clear that by decreasing the value of *H* (getting closer to 0.5) and accordingly the memory of genome, the predictability of DNA walk is decreasing. But in these cases the memory and predictability of DNA walks are decreasing faster than normal DNA walks, which stands for the fact that the damaged DNA is less able to store information and increases its memory. Another difference between plots in Figures 3 and 4 can be seen in the values of the Hurst exponent, where in average the Hurst exponent has smaller values that are closer to *H* = 0.5 in case of damaged DNA compared to normal DNA and this stands for the fact that there is less correlation in the damaged DNA walk compared to normal DNA walk. The averaged value of the Hurst exponent variations for 50 subjects was computed as 0.537 which is smaller than the computed value for healthy subjects.

**Figure 5:**
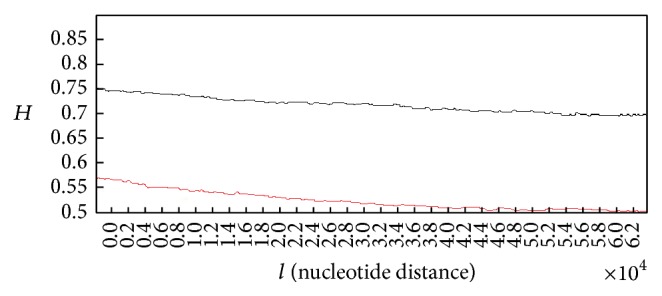
Grand average of the Hurst exponent plots for damaged DNA walks for all of the healthy subjects (black curve) versus grand average of the Hurst exponent plots for damaged DNA walks for all of subjects with lung cancer (red curve).

In order to make a clear comparison, the grand average of the Hurst exponent plots for all of 50 healthy subjects versus the grand average for all of 50 subjects with lung cancer is shown in Figure 5.

The analysis of the grand averaged Hurst exponent plots for the normal and damaged DNA walks gives the results which do not deviate from what have been observed in Figures 3 and 4. As it is clear in case of damaged DNA walk, variations of the Hurst exponent show steeper behaviour than the case that belongs to normal DNA walk. On the other hand, the Hurst exponent has smaller values that are closer to *H* = 0.5 in case of damaged DNA compared to normal DNA.

**Figure 6:**
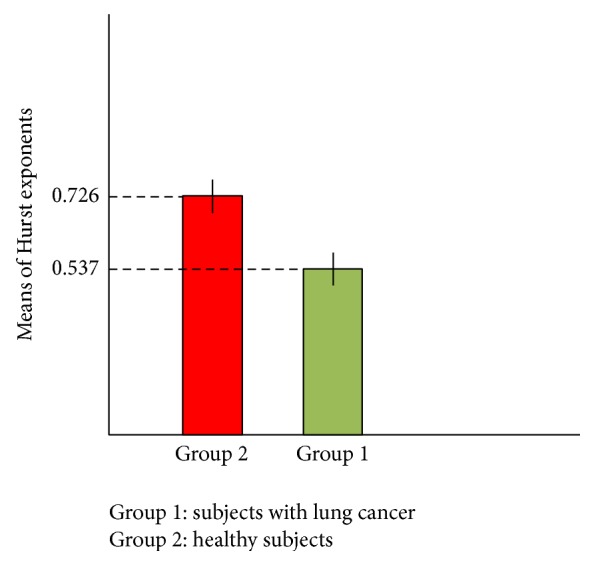
Comparison of confidence interval for means of Hurst exponents.

Also in order to compare the mean of Hurst exponent values in case of each sample we compute 95% confidence intervals in case of healthy subjects and subjects with lung cancer and then determine whether the intervals overlap. As it is known when 95% confidence intervals for the means of two independent populations do not overlap, there will be indeed a statistically significant difference between the means (at the 0.05 level of significance).  Figure 6 shows the computed confidence intervals.

As it is clear in Figure 6, confidence intervals in case of healthy subjects (red bar) with the variation 0.72486 ≤ *X* ≤ 0.72714 and subjects with lung cancer (green bar) with the variation 0.53586 ≤ *X* ≤ 0.53814 do not overlap, which means they are necessarily significantly different. So this result stands for the significant difference between the Hurst exponents values in case of two groups of subjects.

**Figure 7:**
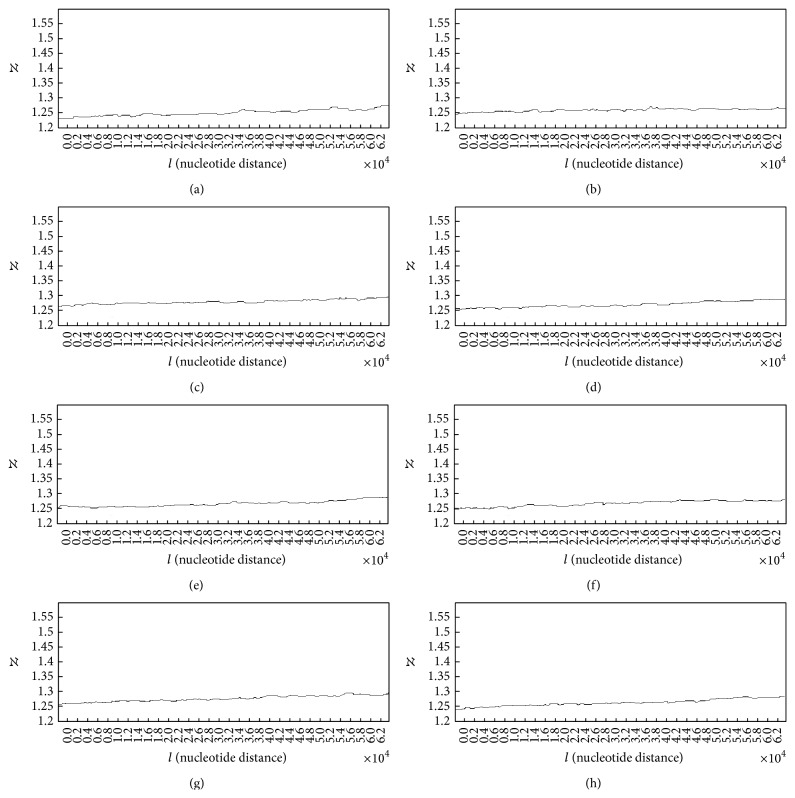
Spectra of fractal dimension plots for DNA walks of 8 healthy subjects.

In order to analyse the complexity of DNA walk in case of normal and damaged DNA walks the fractal dimension spectra of DNA walks are discussed here.  Figure 7 shows the fractal dimension spectra plots for DNA walks in case of healthy subjects.

It is clear in all plots that overall behaviour of the fractal dimension variations is increasing behaviour. This behaviour stands for the fact that complexity of DNA walk is increasing in the genome. The small downward deflections seen in the plots stand for the small decreases in complexity of DNA because of small increases in the genome's memory. By increasing the fractal dimension's value, the predictability of DNA walk is decreasing as the DNA is getting more complex. The averaged value of the fractal dimension variations for 50 subjects was computed as 1.263.

**Figure 8:**
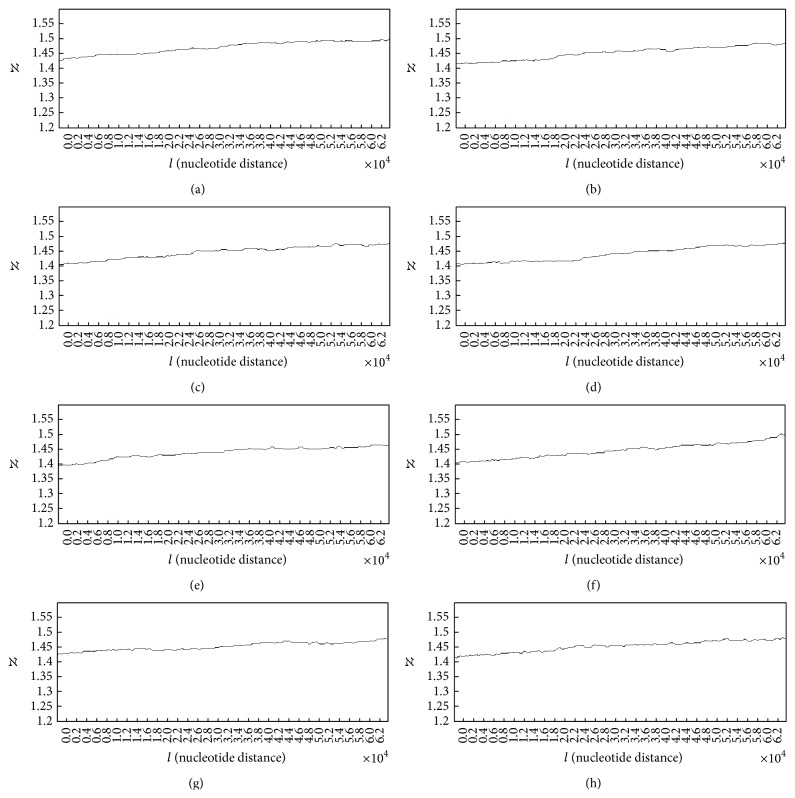
Spectra of fractal dimension plots for damaged DNA walks of 8 patients with lung cancer.

In case of patients with lung cancer some of the spectra of fractal dimension plots for damaged DNA walks are shown in Figure 8.

The analysis of plots for damaged DNA walks shows almost similar behaviour to the fractal dimension plots for the normal DNA walks. As it can be seen in plots (a) to (h) (Figure 8) in overall, the fractal dimension variations show increasing behaviour. But as it is clear, in case of damaged DNA, variations of the fractal dimension show steeper behaviour than cases that belong to normal DNA walks in Figure 7. Like plots in Figure 7, the small downward deflections seen in the plots stand for the small increases in the genome's memory. It is clear that by increasing the value of fractal dimension the predictability of DNA walk is decreasing. But in these cases the predictability of DNA walks is decreasing faster than normal DNA walks. Another difference between plots in Figures 7 and 8 can be seen in the values of the fractal dimension, where in case of damaged DNA the fractal dimension has bigger values compared to normal DNA and this stands for the fact that the damaged DNA walk is more complex compared to normal DNA walk. The averaged value of the fractal dimension variations for 50 subjects was computed as 1.442 which is bigger than the computed value for healthy subjects.

**Figure 9:**
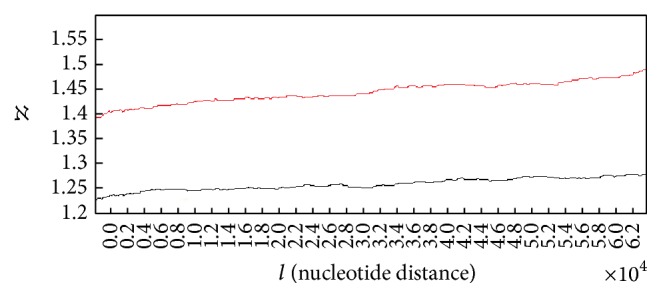
Grand average of the spectra of fractal dimension plots for damaged DNA walks for all of the healthy subjects (black curve) versus grand average of the spectra of fractal dimension plots for damaged DNA walks for all of the subjects with lung cancer (red curve).

In order to make a clear comparison, the grand average of the spectra of fractal dimension plots for all of the 50 healthy subjects versus the grand average for all of the 50 subjects with lung cancer is shown in Figure 9.

The analysis of the grand averaged fractal dimension plots for normal and damaged DNA walks gives the results which do not deviate from what have been observed in Figures 7 and 8. As it is clear in case of damaged DNA walks, variations of the fractal dimension show steeper behaviour than the case that belongs to normal DNA walks. On the other hand, the fractal dimension has bigger values in case of damaged DNA compared to normal DNA.

**Figure 10:**
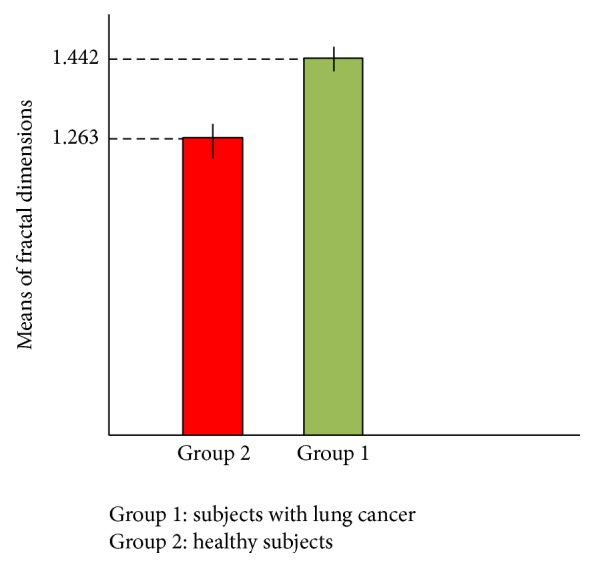
Comparison of confidence interval for means of fractal dimensions.

Also in order to compare the mean of fractal dimension values in case of each sample, we compute 95% confidence intervals in case of healthy subjects and subjects with lung cancer and then determine whether the intervals overlap. As it is known when 95% confidence intervals for the means of two independent populations do not overlap, there will be indeed a statistically significant difference between the means (at the 0.05 level of significance).  Figure 10 shows the computed confidence intervals.

As it is clear in Figure 10, confidence intervals in case of healthy subjects (red bar) with the variation 1.26150 ≤ *Y* ≤ 1.26450 and subjects with lung cancer (green bar) with the variation 1.44060 ≤ *Y* ≤ 1.44340 do not overlap, which means they are necessarily significantly different. So this result stands for the significant difference between the fractal dimensions values in case of two groups of subjects.

All the analyses which have been done in this research showed that by computing the values of the Hurst exponent and fractal dimension we are able to diagnose the damaged DNA's as they show more complexity and less predictability compared to normal DNA's.

## 7. Conclusion

In this paper, we worked on diagnosis of lung cancer by analysing the damaged DNA. By defining the Hurst exponent and fractal dimension we talk about predictability and complexity of damaged DNA. The analyses of Hurst exponent and fractal dimensions plots show that DNA walks have smaller values of the Hurst exponent and bigger values of fractal dimension in case of the damaged DNA compared to normal DNA. Also, the Hurst exponent and fractal dimension plots for damaged DNA show steeper behaviour than normal DNA plots. These results stand for the fact that damaged DNA is less predictable and more complex compared to normal DNA. The method used in this research can be applied for analysis and diagnosis of other types of cancer. Analysing the DNA walk by this method can guide us in modelling and prediction of DNA walk using fractal models.

## Figures and Tables

**Table 1:** Characteristics of movement for each nucleotide.

Nucleotide	Number of occurrence (bp)	Probability	Movement
A	9521	0.3015	−1
G	6398	0.2026	−1
T	9487	0.3004	1
C	6170	0.1954	1
Total value	31576	1	

**Table 2:** Some published values of *H* for DNA sequences.

Sequence	*H*	Reference
Human beta-cardiac myosin Heavy chain gene	0.67	[25]
Human beta-globin gene	0.708	[26]
Synthetic model sequence	0.655	[26]
